# The Limitations of Being a Copycat: Learning Golf Putting Through Auditory and Visual Guidance

**DOI:** 10.3389/fpsyg.2019.00092

**Published:** 2019-02-01

**Authors:** Marta M. N. Bieńkiewicz, Lionel Bringoux, Franck Buloup, Matthew Rodger, Cathy Craig, Christophe Bourdin

**Affiliations:** ^1^Aix-Marseille Université, CNRS, ISM, UMR 7287, Marseille, France; ^2^School of Psychology, Queen's University of Belfast, Belfast, United Kingdom; ^3^INCISIV Ltd, Belfast, United Kingdom; ^4^School of Psychology at Ulster University, Coleraine, United Kingdom

**Keywords:** auditory-visual perception, motor learning and control, movement guidance, golf putting, kinematic template

## Abstract

The goal of this study was to investigate whether sensory cues carrying the kinematic template of expert performance (produced by mapping movement to a sound or visual cue) displayed prior to and during movement execution can **enhance motor learning of a new skill (golf putting)** in a group of novices. We conducted a motor learning study on a sample of 30 participants who were divided into three groups: a control, an auditory guide and visual guide group. The learning phase comprised of two sessions per week over a period of 4 weeks, giving rise to eight sessions. In each session participants made 20 shots to three different putting distances. All participants had their measurements taken at separate sessions without any guidance: baseline, transfer (different distances) and retention 2 weeks later. Results revealed a subtle improvement in goal attainment and a decrease in kinematic variability in the sensory groups (auditory and visual) compared to the control group. The comparable changes in performance between the visual and auditory guide groups, particularly during training, supports the idea that temporal patterns relevant to motor control can be perceived similarly through either visual or auditory modalities. This opens up the use of auditory displays to inform motor learning in tasks or situations where visual attention is otherwise constrained or unsuitable. Further research into the most useful template actions to display to learners may thus still support effective auditory guidance in motor learning.

## Highlights

- Auditory guidance can influence motor learning processes in a way that is similar to a visual motion display.
- Sensory guidance leads to dependency on the display as performance drops when the display is no longer available.
- Biomechanical and individual differences were not considered, but might be a key to the successful design of sensory feedback.
- Concurrent feedback might have a different impact on motor learning than a guidance (“copycat”) approach.

## Introduction

Motor learning can be described as a lasting improvement in performance compared to a baseline measure that can be attributed to training (Shmuelof et al., 2012; Sigrist et al., 2013). Fitts and Posner (1967) described motor learning processes as passing through three stages: from the first stage of very attentive and effortful movement, to the second stage of fine tuning of the action to the final stage of automation, or at least partial automation, of the movement. When a skill is mastered we observe successful goal attainment, but also reductions in the variability of movement across repetitions and an increase in movement smoothness. Those mechanisms provide evidence for efficient feedback control mechanisms (Shmuelof et al., 2012), which allow the performer to fine-tune previously performed movements at the next opportunity (Yousif and Diedrichsen, 2012). For example, in a golf swing study by Lai Ab et al. (2011) skilled golf players demonstrated more consistent swing patterns in their pelvis movements than beginners. In this study we examined the effects of sensory guidance on motor learning in a golf putting task. We assessed levels of motor learning by measuring both putting success and reductions in variability, which may be independent of each other when refining putting technique (Richardson et al., 2018).

Contrary to the popular belief that a fixed number of hours are required to learn a new skill, research has shown that the speed with which people learn will depend on both practice effort and personal abilities (Hambrick et al., 2014). For example, learning how to play golf, like any other complex motor behavior, is effortful, prone to error and frustration, and requires external guidance to efficiently control the different kinematic parameters. Teachers and coaches use a variety of methods to facilitate learning. Verbal instruction is usually given along with a visual demonstration of the movement from another person (usually a coach). The coach will also offer further instruction on which specific aspects of the movement the player needs to focus on to improve performance. However, verbal instruction alone is not sufficient to improve performance in complex skills like golf putting. For instance, a posteriori verbal instruction seems inappropriate and too non-specific to guide the desired timing of learner's movements to create an “ideal” putt.

### Describing the Golf Putting Action

**Figure 1:**
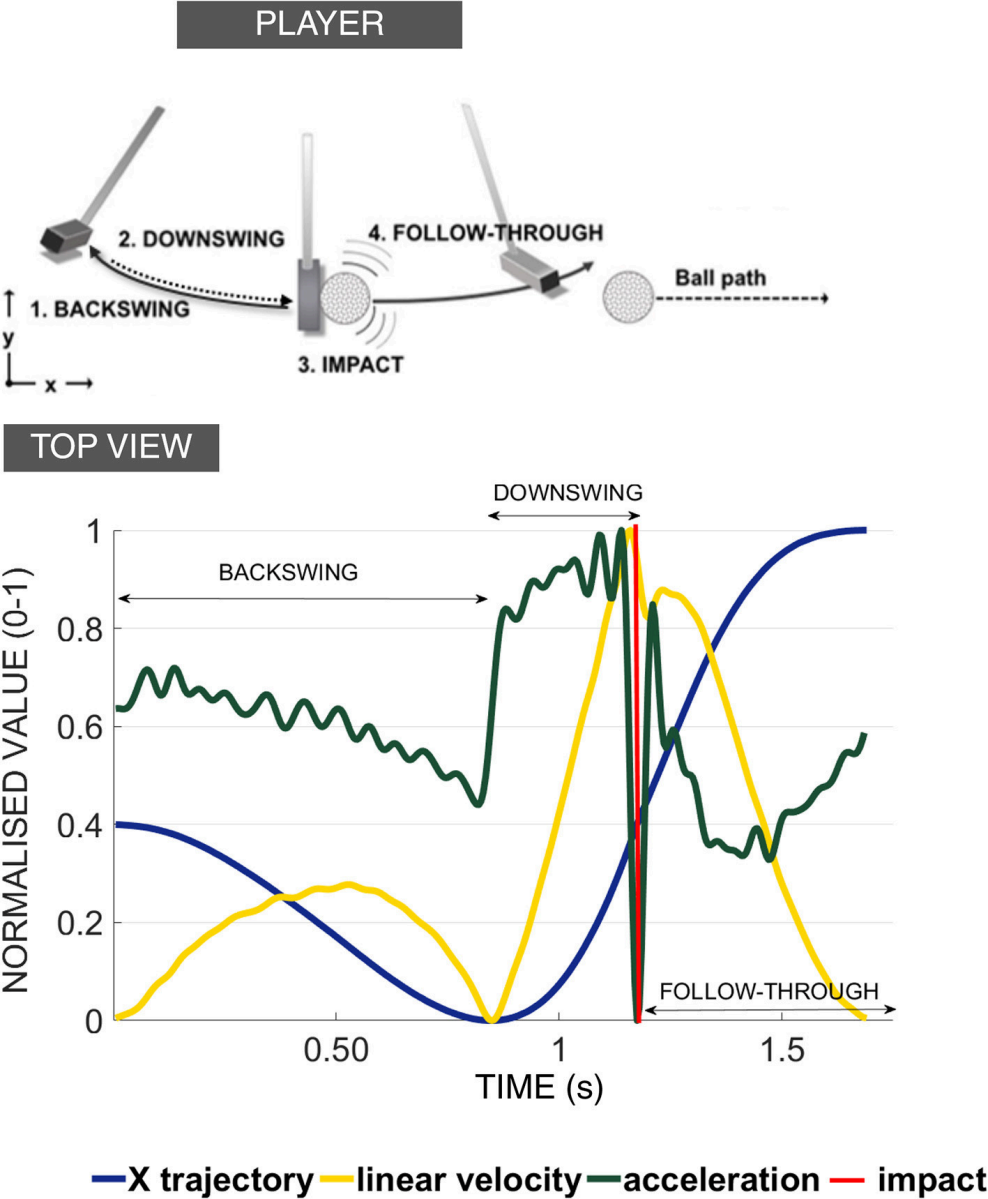
Four phases of golf putting action. Diagram illustrating the four phases of golf putting action. These phases are defined as follows: **Backswing:** when the player moves the club from the starting point away from the ball; **Downswing:** when the player moves the club from the endpoint of the backswing toward the ball; **Impact:** when the club and the ball make contact (approx. 30 ms, Burchfield and Venkatesan, 2010); **Follow-through:** when the club continues to move after the impact. The top panel depicts changes in direction from the endpoints of each phase (in this example it represents a leftward putt). The bottom panel depicts the kinematic characteristics of each phase of a successful 6 m putt made by the professional player. The point of impact is demarcated with a red vertical line. Note how the backswing duration is more than double the duration of the downswing (in this example temporal ratio of 2.5:1).

The putting action can be broken down into four principal phases: backswing, downswing, impact, and follow through (see Figure 1). There are a few major factors that have been found to be linked to the consistency and repeatability of the golf putting swing: namely movement velocity, velocity through the swing motion path immediately surrounding impact and the temporal ratio of the backswing to the downswing (Burchfield and Venkatesan, 2010). The ideal ratio is considered to be 2:1 (backswing phase being twice as long as the downswing) (Grober, 2009; Kooyman et al., 2013) regardless of the target distance of the putt (Grober, 2011). Other non-golf related studies have demonstrated that the human motor system generates a spontaneous movement tempo to use the least force to generate motion (Bove et al., 2009; Avanzino et al., 2015; Bisio et al., 2015). The “ideal ratio” was found to lead to good control improving the accuracy and distance of the putt. The ratio also allowed the random errors caused by the magnitude of the applied forces to be minimized and the velocity of the club head at ball impact to be kept relatively constant^1^ (Grober, 2009). Players can feel their natural tempo by swinging the club back and forth and are often observed doing it almost instinctively before hitting the ball. In a study by Kooyman et al. (2013), it was found that golfers who received visual feedback on their temporal ratio of their putting action over three different putting distances using a custom-made GUI, improved their putting motion and decreased shot variability for both the experienced and inexperienced golfers.

Accurate golf putting requires that a golfer exhibits the finest degree of control of both the spatial and temporal parameters of the movement. The putter allows for the efficient transfer of energy generated by the movement dynamics of the golfer to the ball so that it travels the required distance. It is important to note, however, that this is the case only if the center of the putter face hits the ball. Golfers who showed high levels of putting ability were found to show reduced variability of the movement (Burchfield and Venkatesan, 2010). The seminal study by Craig et al. (2000) found a linear relationship between the putting distance and clubhead velocity at ball impact.

### Feedback and Motor Learning in Golf

Based on the features mentioned above, we chose a golf putting task as an example of a complex motor task. The aim of this study was to see if sound could be used to convey the dynamics of an expert's motion and help accelerate the learning of a putting task in a group of novices. There is a growing body of research that is examining the efficacy of sensory guidance and action observation to improve motor performance, which has relevance not only for sport, but also for the recovery of motor function (Krakauer, 2006). When using sensory guidance, the learner is presented with a template which provides information about how to perform an action. This approach differs from augmented feedback that is directly connected to the learner's own movement (see Sigrist et al., 2013 for a review).

In the context of providing sensory guidance to enhance motor learning in golf putting, it is mandatory to consider the specificity of the skill to be learned. In golf, instructors ask players to keep their eye on the ball whilst swinging the club. Such instructions make it difficult to use visual guidance to improve movement as following a visual guide would compromise the ability to focus their visual attention on the target that needs to be hit. In this study, we decided to examine the difference between the efficacy of auditory information compared to visual information as a way of helping novices improve their performance in a golf putting task.

An auditory signal can provide information about club-head velocity and the temporal ratio of the backswing to the downswing (Murgia et al., 2012), allowing the golfer to visually attend to the spatial aspects of the task (i.e., assessing the putt distance and keeping their eyes on the ball whilst swinging the club). We transformed movement data into auditory information, using a process called “movement sonification” (defined in broad terms as the mapping of movement data onto pre-defined sound parameters). Sound may not only be more effective for conveying temporal information than vision (Hirsh and Watson, 1996; Murgia et al., 2017), but also uses fewer attentional resources and is more portable (Secoli et al., 2011). A few studies have already demonstrated that sonification can be used to guide motion in simple tasks. Young et al. (2014) found that both healthy controls and Parkinson's disease patients are able to re-enact step lengths from recorded sounds of the footsteps of a neurologically intact person when walking on gravel. Both groups were able to adapt their gait irrespective of whether they heard actual sounds or recalled them from memory. This study provided evidence that sound is a powerful carrier of the kinematic features of movement, at least for this clinical population. Interestingly, the actual information that was relayed by the environmental sound (natural recordings) was reported to be a richer source of information than the synthesized sounds, possibly due to the fact they depicted the motor action in a more holistic (*Gestalt*) way (Koffka, 1999; Kennel et al., 2014). A similar effect was observed by Murgia et al. (2016) when they studied the natural recording of breathing sounds vs. engineered sounds conveying the same temporal structure. Improving motor behavior (learning) by employing auditory displays has also been reported in sports related contexts. For example, Agostini et al. (2004), designed an experiment where athletes were performing hammer throws while listening to the natural recording of their best throw the previous day. It led to increase in the throw length and a decrease in the throw variability. Schaffert and Mattes (2014) used augmented acoustic feedback from a boat's acceleration-time trace in a rowing experiment with high-performance squads. The presence of an auditory display enhanced mean boat speed when compared to the baseline performance of each squad and immediate retention effect was also present after the withdrawal of the feedback. In addition, athletes reported auditory feedback as beneficial in providing additional information to supplement the already available visual feedback relating to their performance. A study by Effenberg et al. (2016) demonstrated that four dimensional sonification of rowing movement parameters (grip force, sum of footrest forces, grip pull-out length, and sliding seat position) with a modulation in frequency and amplitude (combined with video instruction and recording of sonification from an expert) can enhance motor learning. The effects observed with sonified stimuli were beyond enhancement observed with the use a pacemaker sound, or natural sound guidance in comparison. Interestingly, the effects were still present at a 3-week retention measurement test.

### Sensory Guidance and Motor Learning: A Theoretical Perspective

In terms of trying to understand why sensory guidance may help skill acquisition, a variety of different yet converging perspectives have been put forward. From an ecological psychology perspective, motor skill acquisition can be defined as an improved use and handing of informational variables available in the environment (Jacobs and Michaels, 2007; Huys et al., 2009; Gray, 2010; Huet et al., 2011). In that sense, novices can be described as perceivers with pre-existing skills for perception and action learning who adapt their performance in response to training of their attention (Dyer et al., 2017). Alternatively, the concept of perception guiding action can be referred to as a feed-forward model of human motor control. Humans are believed to internally simulate the movement prior to execution and then correct it during action performance based on feedback (external and proprioceptive) (Wolpert et al., 1995). The same feed-forward can be applied to conceptualizing what happens when our own actions are organized with regards to external movement patterns, both biological or non-biological (de Wit and Buxbaum, 2017). In other words, our brain is designed for perception to guide and correct action, but also to understand the actions of others via the same neural networks (Rizzolatti and Craighero, 2004). Regardless of different theoretical approaches that link perception and action, neuroimaging studies show that humans exhibit an affinity for human velocity patterns in motion (Stadler et al., 2011, 2012), even if it is reduced to a display comprising of a few points of light (Johansson, 1973). Moreover, the detection of patterns of human action is likely a “supramodal” process, that is, independent of whether the movement is perceived visually or auditorily (Rosenblum et al., 2017).

Many studies show that visual guidance can facilitate motor learning of a new skill. In a study investigating the effects of observational learning on golf swing performance in a group of novices, the results showed that participants benefited when their attention was being visually guided to specific aspects of the movement (D'Innocenzo et al., 2016). Directing attention straight to accentuated points in the display was more beneficial than observing the movement of an expert alone or replaying their own performance on a video recording. Another study that looked at the effects of observational learning when learning to bowl a delivery in cricket, found that point light displays improved interlimb coordination during the movement and helped participants recreate a movement that resembled the model movement in the full body display (Breslin et al., 2009). Similar results were reported for video and point light displays in learning to kick a soccer ball in a group novices. Results again showed that there was a convergence toward the kinematics demonstrated in the model movement, without any impact on success or accuracy of the kicks (Horn et al., 2002).

### Research Questions

**Figure 2:**
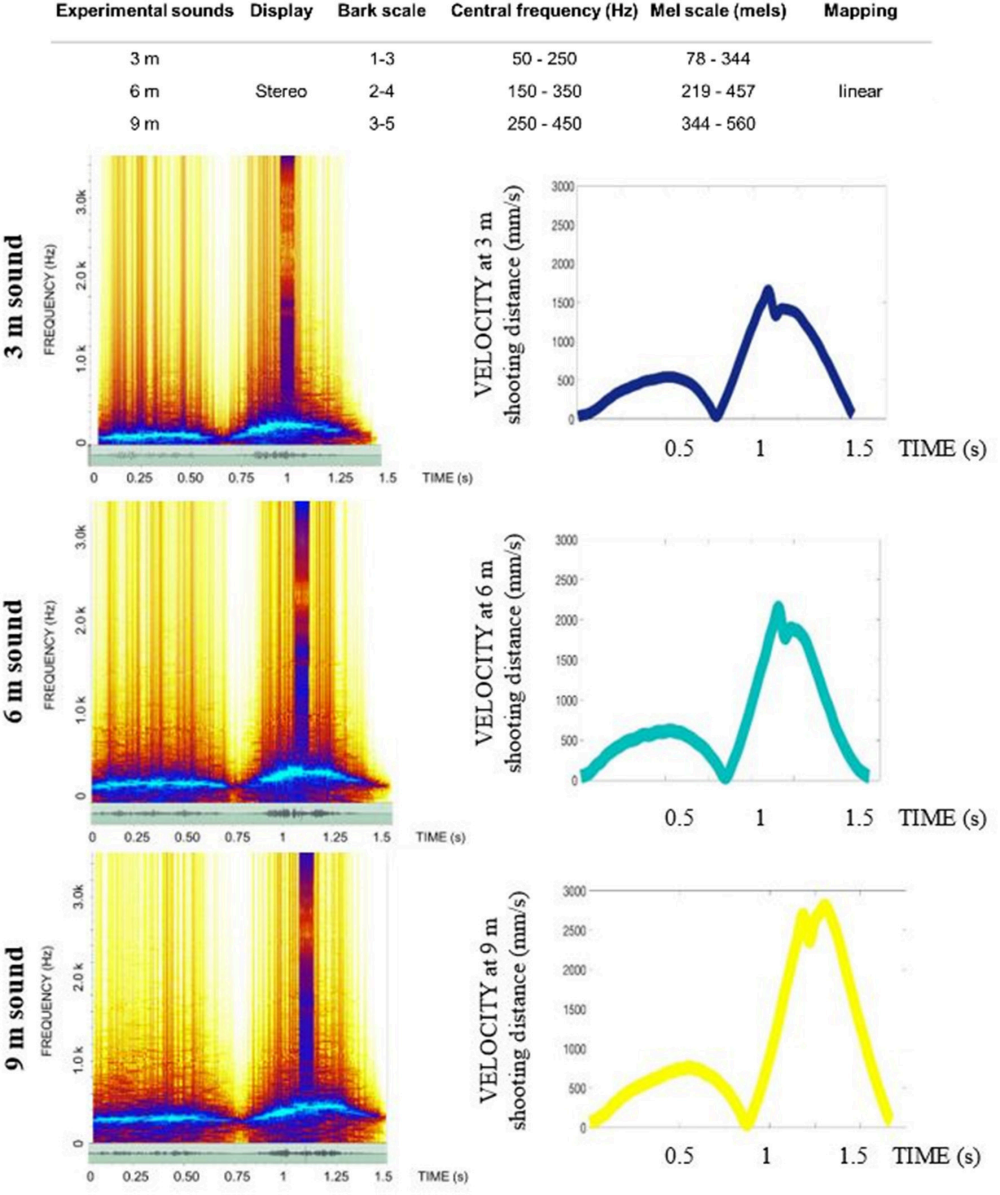
Sound stimuli for the GS condition. The spectograms of the sound stimuli used in the generation of the GS with the original velocity curves derived from the motion capture recordings of the professional player (for 3, 6, 9 m successful putts). See Supplementary Information to listen to the sounds used in the Experiment.

**Figure 3:**
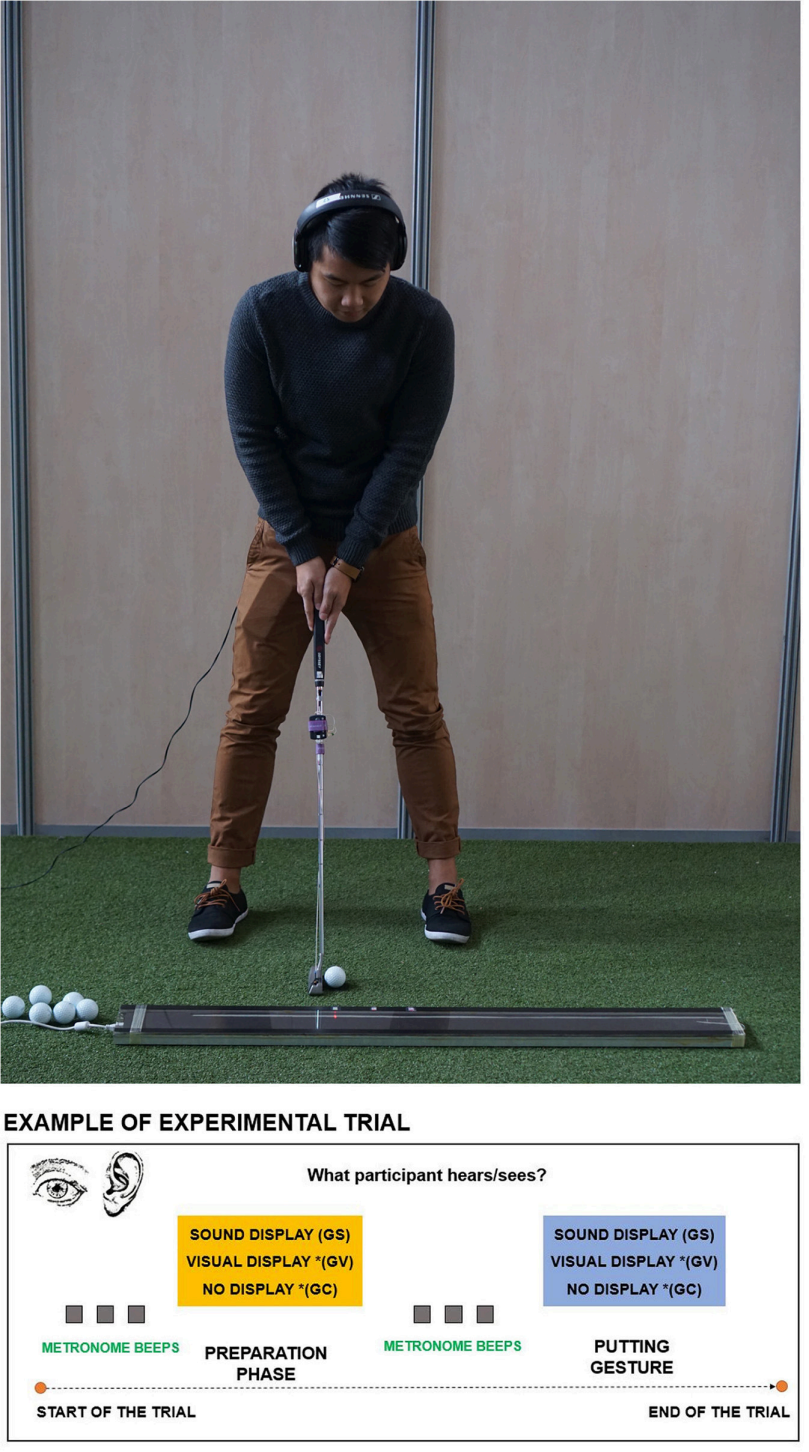
An illustration of the visual display. **(Top)** A subject from the GV in the process of learning using the visual guide (*written informed consent was obtained from the depicted individual for the publication of this image*). The ball is aligned to the starting position of the display. The participant waits for the display to launch, observes the first loop of the display and then moves along with the second loop. **(Bottom)** Flowchart depicting experimental procedure in each trial respective of participant's group. See **Videos 1, 2** to see LED guide used in the Experiment.

The core research question in this study is to investigate whether people can achieve better learning outcomes if a perfect “copy” of the movement dynamics and tempo is made available to them via an auditory channel. We call this approach the “copycat” approach as it aims to imitate someone else's behavior. Our idea is based on how skills are learned in real-life settings: people often try to track a particular motion template, or rhythm, presented in a single sensory domain—usually visual. Occasionally coaches haptically guide the movement of students by using their own motion to convey the template information via the proprioceptive channel. In this study, we adopt a novel approach, where a novice is presented with an expert's kinematic template of movement that is encapsulated in patterns of sound. This sound contains temporal information to guide movement just before (feedforward) and concurrent to the execution. In doing so, both the relative spatial and temporal characteristics of the movement are conveyed via sound so they can be re-enacted (Young et al., 2013). To explore this novel approach, we recorded the putting performance of a professional golfer when putting to three distances to provide the kinematic pattern for both an auditory and visual display (Figures 2, 3, respectively) that could be used later to assist learning in groups of students learning to putt a golf ball.

We posed three research questions:

1. Does auditory sensory guidance improve learning in terms of goal attainment (spatial accuracy of golf putts - number of hits and distance to the hole)?
2. Does auditory sensory guidance improve learning in terms of reduced kinematic (impact velocity) and timing (temporal ratio) variability of the putting movement?
3. How does auditory guidance differ from visual guidance when learning to putt a ball to predefined distances?

## Methods

### Participants

Thirty right-handed Sport Science students at Aix-Marseille University took part in the experiment (mean age:19.6 ± 2.4 years). Participants were asked not to take up any golf related practice outside of the training for the duration of the study. None of the participants had previous experience playing golf or putting. All participants had normal or corrected to normal vision and no hearing impairments. All participants provided written and informed consent to voluntarily participate in the study, in exchange for student course credits. All participants were informed of their right to withdraw at any time. This study was performed in accordance with the ethical standards of the Declaration of Helsinki (Salako, 2006). The protocol was approved by the Ethics Committee of Aix-Marseille University.

### Protocol

After the baseline measurements were collected from all participants (ten putts to three distances: 3, 6, 9 m), they were pseudo-randomly assigned to one of three experimental groups (*n* = 10) such that there were two females per group (mean age Control: 19.9 ± 2.2, Sound: 20.1 ± 3.1, Visual: 19.3 ± 1.8 years). The three experimental groups were:

- Control Group (GC) – learning to putt with no sensory guidance
- Sound display Group (GS) – learning to putt with auditory guidance
- Visual display Group (GV) – learning to putt with visual guidance

The number of sessions and timeline of the study is depicted in Table 1. Participants were asked to train by putting a golf ball a certain number of times (as determined by the session requirements) to each of the distances (See Table 1). During the learning sessions, the first five putts were made to each target distance and were recorded as retrieval trials (i.e., performed without any sensory display (sound or vision) being made available). A further fifteen putts were also recorded as learning trials where the sound or visual display was made available to the GS and GV respectively, with no display for the GC. The order of putting distances was randomized in each session using custom made software (Docometre).

**Table 1:** Presentation of the study design and time schedule.

**Groups**			**GS and GV**		**GC**	
**Week**	**Session**	**Distances**	**Display**	**Nr of trials per distance**	**Display**	**Nr of trials per distance**
W1	BS		No	10		10
W2	LS1		No	RT: 5		RT: 5
			Yes	PT: 15		PT: 15
	LS2		No	RT: 5		RT: 5
			Yes	PT: 15		PT: 15
W3	LS3		No	RT: 5		RT: 5
			Yes	PT: 15		PT: 15
	LS4	3/6/9 m	No	RT: 5	No	RT: 5
			Yes	PT: 15		PT: 15
W4	LS5		No	RT: 5		RT: 5
			Yes	PT: 15		PT: 15
	LS6		No	RT: 5		RT: 5
			Yes	PT: 15		PT: 15
W5	LS7		No	RT: 5		RT: 5
			Yes	PT: 15		PT: 15
	LS8		No	RT: 5		RT: 5
			Yes	PT: 15		PT: 15
W5	TS	4.5/7.5 m	No	10	No	10
W6–W7			BREAK			
W8	RS	3/6/9 m	No	10	No	10

*GC, control group; GS, group with sound display; GV, group with visual display, BS, Baseline Session; LS 1-8, Learning Sessions; RS, Retention Session; TS, Transfer Session; RT, Retrieval Trials; PT, Practice Trials*.

Participants performed 120 practice shots to each putt length (360 in total across three lengths) with 40 retrieval trials (120 in total across three lengths) over eight learning sessions (4 weeks). The breakdown of each session is available in Table 1. Baseline measurements were conducted 2 weeks prior to the start of the training and the retention measures were taken 2 weeks after the end of the training. Transfer tests were conducted immediately after the last learning session (8th) for each participant and comprised of two new putting distances: 4.5 and 7.5 m.

Each trial had two phases (see Figure 3, bottom panel). The first was a preparation phase where the participant was instructed to focus on the ball and the putting distance, and second was a putting gesture phase where the participant was instructed to hit the ball as soon as s/he felt ready. Each phase was preceded with three metronome beeps (60 bpm, 500 ms inter-beep-duration, 440 Hz) to control the general timing instructions to putt in each trial. Participants were instructed to move after the last beep of the metronome in the Gesture putting phase. For the GS and GV participants, they either listened to the sound or observed the LED display after three metronome beeps. In the GC and GV a continuous pink noise (duration matching sound duration in GS for each length) was played after each metronome display to match the presence of sound in GS. In the Baseline, Transfer and Retention tests for all of the groups (GC, GS, GV) they performed the shots with a metronome followed by a continuous pink noise (duration 1.5 s).

### Apparatus

**Figure 4:**
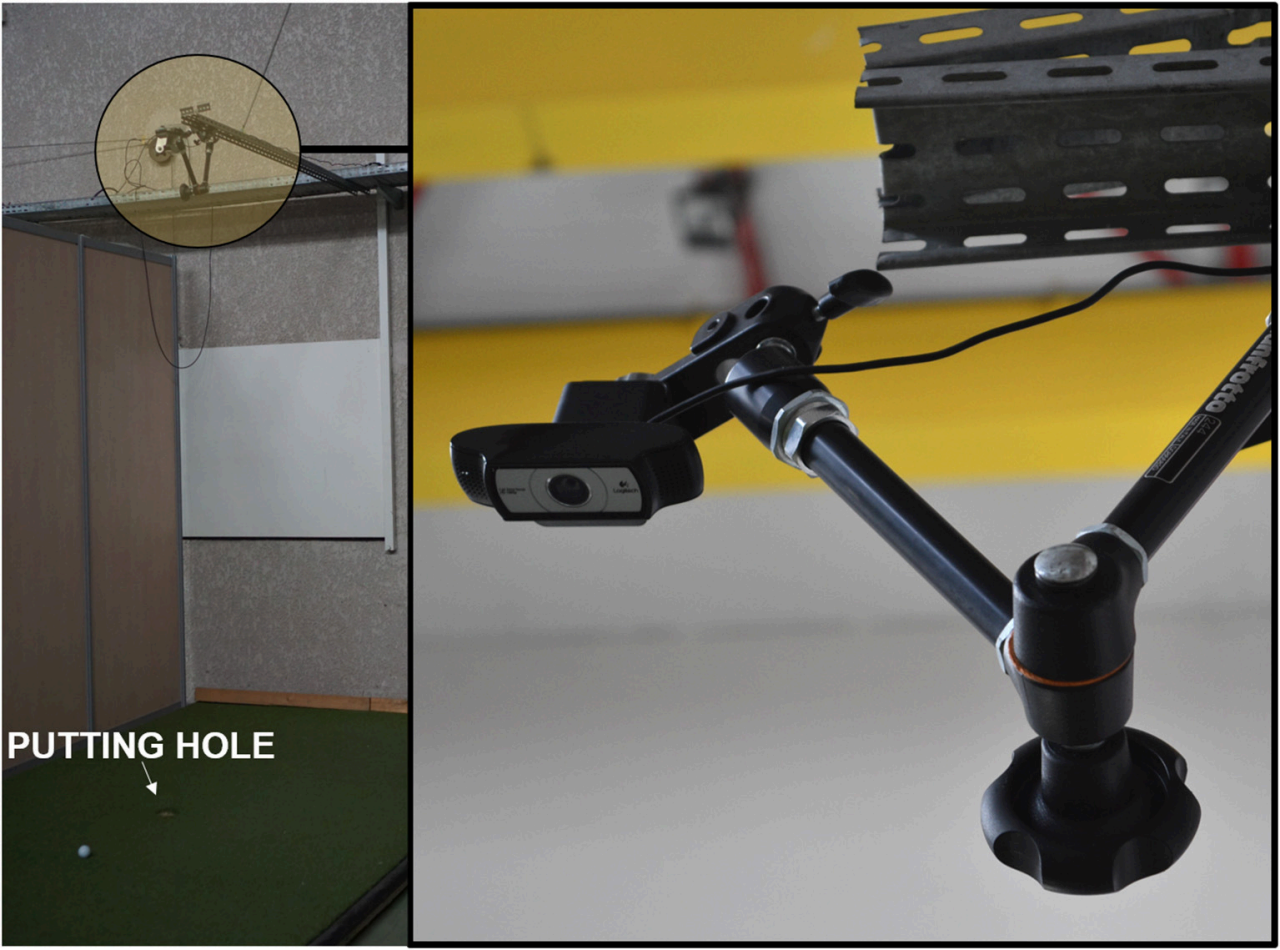
Camera set up in the lab. Camera was set up parallel to the putting green directly over the putting hole (2.5 m above).

A 2 × 0.03 × 15 m (W × H × L) artificial golf green was positioned on wooden planks with a 10.8 cm hole cut out 1.5 m from the wall end in a dedicated golf putting lab. Five black painted marks on the artificial green were made to determine five distances to the hole (**3**, **4.5**, **6**, **7.5**, **9 m**). Although we chose three distances (**3**, **6**, **9 m**) to manipulate the difficulty of the task, we are aware that the typical putt in the game of golf does not normally exceed 7.5 m (Burchfield and Venkatesan, 2010). A Logitech Camera (HD Video Camera- Pro Webcam C930e) was mounted on an extended mechanical arm parallel to the green and overlooked the putting hole (2.5 m above the putting green) and allowed us to measure the accuracy of each putt (Figure 4). The camera was controlled using custom-made software that recorded ball movement at 30 Hz. The recording was triggered at the start of each trial and was stopped by the researcher when the ball was stationary near the hole. An Oddysey White Ice putter for right handers was used for the task, along with a set of Titleist balls PROV1X (60 balls for each session). All putting movements were recorded using the CodaMotion system. One CX1 camera was placed parallel to the starting position of the putt on the putting green, with infra-red active markers being placed near the top of the putter shaft and on the club head of the putter. Positional data of the movement of the putter were exported to Matlab for processing. The launch of trials and all the devices connected were controlled using the Adwin Gold system (©JAGER GmbH) piloted via our in-house Docometre software. Sound was delivered by a Raspberry Pi and custom-made program based on the ALSA software. Participants in all groups were wearing Sennheiser headphones to provide them with an auditory cue to signal the launch of the trials.

### Design

#### Copycat Approach

For the GS and GV, we designed the sensory displays based on the performance of an expert golfer (copycat approach). To do so, we invited a professional player to putt a golf ball to three distances (3, 6, 9 m) during the pilot stage of this study and recorded his movement using the CodaMotion motion capture camera CX1 and two infra-red active markers placed near the top of the putter shaft and the club head (see Table 2). The sound of ball impact was also recorded with a portable microphone (ZOOM H4 handy microphone) placed on the putting green 15 cm from the golf ball at each putting distance. We chose the best putt across the expert golfer's successful trials (ball going in the hole), based on the visual inspection of the velocity curve and personal feedback from the player. We chose the first derivative of the spatial position to create the pattern of information presented in the sensory displays—auditory (GS) and visual (GV) and also determine the time of ball impact in the action.

**Table 2:** Summary of characteristics for the professional player movement for the trials that were used for design of acoustic and visual displays.

**Parameters**	**3 m**	**6 m**	**9 m**
Backswing amplitude (radial)	12.32	17.55	19.96
Downswing amplitude (radial)	13.27	18.41	20.79
Backswing metric amplitude (mm)	267.8	339.5	412.1
Downswing metric amplitude (mm)	261.4	340.2	406.1
Backswing peak velocity (mm/s)	539.4	620.4	762.4
Backswing mean velocity (mm/s)	343.3	410.1	488.7
Backswing STD of velocity (mm/s)	168.2	181.1	218.9
Downswing peak velocity (mm/s)	1,656.9	2,149	2,691.7
Downswing mean velocity (mm/s)	864.9	1,039.7	1,278.8
Downswing STD of velocity (mm/s)	522.9	690.3	859.8
Velocity at impact (mm/s)	1,601.9	2,018.9	2,603.9
Temporal ratio MT backswing/ MT downswing	2.57	2.54	2.66

*MT, movement time; STD, standard deviation*.

#### Auditory Guidance for GS

Many studies select a sonification method a priori without considering what is important for the design of the sound stimuli (Sigrist et al., 2013). In fact, there is a need for research to map properties of sound, such as amplitude, brightness, or loudness, onto movement parameters. To convey the motion in sound in the best possible way, we ran two pre-tests to decide on the best sound design to use (see O'Brien et al., 2018). The sounds implemented in this study (Figure 2) were synthesized using a tailor-made script as white noise with the center of a band-pass filter mapped to velocity (“whoosh” sound designed to resemble the aural consequence of metal club cutting through the air). We used a psychometric conversion to the Mel scale incorporating a linear mapping of the velocity signal. We added a stereo effect reflecting the positional changes of the golf club with respect to the midline axis of the body. To convey the changes in the energy levels necessary to putt to longer distances (effectively increasing the movement velocity) the sound for the **3 m** putt was scaled on a band from 56 to 252 Hz (with peak velocity of the movement of the pro player being 0.56 of the value of the **9 m** peak velocity); the **6 m** putt was scaled on the band of 158–358 Hz (with peak velocity of movement of the pro player being 0.80 the value of the **9 m** peak velocity); and the **9 m** putt was scaled to 250–450 Hz. The pre-recorded sound of impact was embedded into the sound to correspond to the point of impact between the club and ball and was based on the kinematic recordings.

#### Visual Guidance for GV

To depict motion visually, we used a LED guide consisting of a series of 400 linearly aligned LEDs (1.2 m long) fully programmable and mounted in a portable, rectangular unit, with a PIC board inside (Figure 3, top panel). The connection was set up via a PCB USB adapter to the external computer, which allowed us to trigger the display using a User Datagram Protocol (UDP) predesignated signal (Unicode character). The custom software made in C++ meant we could load any artificial, biological motion profile allowing us to control the number of LEDs involved in the display and the time each was lit for. Using a custom-made script in Matlab, we translated the position on the x axis into the LED display scaling the amplitude of the movement to the amplitude of the display (see Table 2 for information on speed and amplitude of movements across different putt distances). The congruency between the display and the original kinematic was previously validated using video tracking method in a prototype of the used LED guide in a study by Bienkiewicz et al. (2013). The original motion capture profile of the expert golfer was translated into the LED display using a custom MATLAB script that translated positional data into the amplitude and time that each LED was lit up for. This programme has full functionality to determine the direction and the timing of the LED display. This way, the visual motion of the expert player was depicted as a point of light moving in a linear fashion on a predesignated path conveyed the movement of a club head in a golf putting movement (See Video 1).

We validated the span of the display with the actual physical measurements of the swing from the motion capture and observed differences of ± 5 mm due to the small gaps between blocks of LEDs. The UDP character was sent via a LAN connection to launch the guide in sync with the other devices.

#### Calibration Method for Video Acquisition

For each participant, and each trial, camera images from the experimental sessions were captured at a frequency of 30 Hz and saved in a separate folder. Post session, all images were processed using the automatic custom-made ball trajectory recognition software Eclipse RCP and OpenCV technologies. Algorithms were able to detect the contrast between the background putting green and the ball, tracking the point that corresponded to the center of the ball. The coordinates of the ball in each frame were extracted and saved as a text file. Each trial was visually inspected to verify that the automatic tracking was correct. If there was too much light or an alien object was present in the camera view distorting the recognition, relevant masks were applied and the trial was reprocessed.

The video calibration was applied to the post-processed text files to translate the pixel coordinates into the physical metric coordinates of the experimental space. This was done using a custom written Python script that incorporated static and dynamic calibration using an A3 print-out of a chessboard panel (calibration image). Firstly, the camera's intrinsic parameters and distortion coefficients were computed using 32 images taken at different perspectives. This allowed us to transform the image obtained using coefficients that could account for the light modification due to hardware properties. Secondly, perspective projection was computed using homography of a pixel position mapping onto the experimental metric space in a reference calibration image. The origin was placed in the center pixel of the putting hole. After the calibration processing, each trial had a text file with a metric position for the ball in each trial. This data was used for further analysis.

### Data Extraction

Trials where participants failed to smoothly strike the ball (i.e., when two or more peaks were detected in the velocity profile around ball impact point due to the participant hitting the putting green before the ball) were excluded from any further kinematic analysis. We chose to run an analysis that calculated the linear velocity relative to the putt-direction axis rather than the angular velocity as the latter can misrepresent impact dynamics if the movement is not performed by a professional (i.e., if the participant is a novice and has a putting action that does not follow a semi-circular movement path).

For the velocity calculation, we applied a low-pass Butterworth filter of 20 Hz, 8th order based on the RMSE method to ensure minimal data loss of the 20 randomly selected recordings of positional data from the data pool. The beginning of the movement was automatically detected as being when the movement velocity exceeded 2% of backswing peak velocity (x axis), and the end of the gesture was denoted as the point when the velocity fell below 2% of follow-through peak velocity (x axis).

### Statistical Analysis

To explore if there are differences in the way all three groups learned the task, we divided the analysis into three parts: (1) The spatial accuracy of the putts (percentage of successful putts and distance from the hole), (2) Kinematic variability (standard deviation of impact velocity across trials), and (3) Temporal ratio (time spent in backswing movement divided by time spent in downswing movement). To account for the variability in the initial performance between groups we normalized (standardized) the learning sessions and retention data for all of the presented variables to the baseline performance for each individual. For the learning sessions we analyzed separately the retrieval trials (first five shots during the learning sessions, for sensory groups performed without guidance, see Table 1) and practice trials (fifteen putts following retrieval, for sensory groups performed with guidance, see Table 1).

The analysis presented in the results section compares the performance of three different groups of learners over an eight-week period. The learners were divided into three groups and received (i) auditory, (ii) visual or (iii) no sensory guidance when learning to putt a ball in golf.

For all outcome variables, mixed ANOVAs were carried out with group as a between-subjects factor and both target distance and session number as within-subject factors, unless otherwise indicated. Where main effects were detected, *post-hoc* Bonferroni-adjusted *t*-tests were carried out. Where the assumption of sphericity was violated, Greenhouse-Geisser adjustments to degrees of freedom are reported.

To estimate the effect size of factors we used partial eta-squared (η_*p*_^2^) calculations, and complied with the interpretation of indexes proposed by Cohen (1988) (0.01 = small effect; 0.06 = medium effect; 0.14 = large effect). Statistical significance was set at the 5% level.

## Results

### Results Referring to Research Questions 1 and 2

#### Spatial Accuracy of Golf Putts

**Figure 5:**
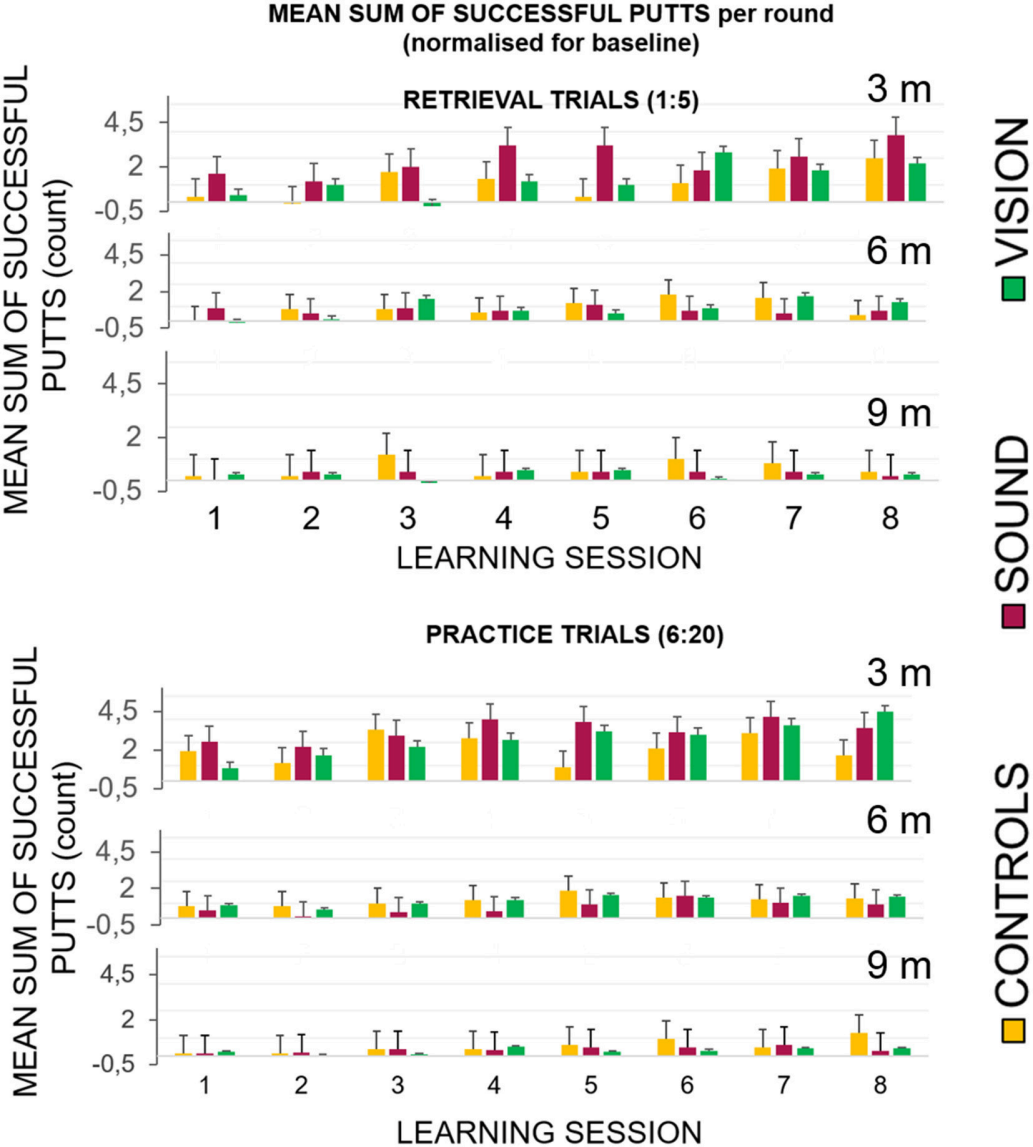
Success rates broken down for each of the learning sessions across the three groups. **(Top)** Success rates for the learning sessions (LS1–LS8) during the retrieval trials (no guidance in GS and GV). The graphs show the first five shots of each session, and the practice trials when the sensory groups had acoustic and visual guides, respectively. All groups performed better with the progression of the sessions. **(Bottom)** The GV condition had a visible dissonance effect between the retrieval and practice conditions suggesting a greater level of sensory dependency.

**Figure 6:**
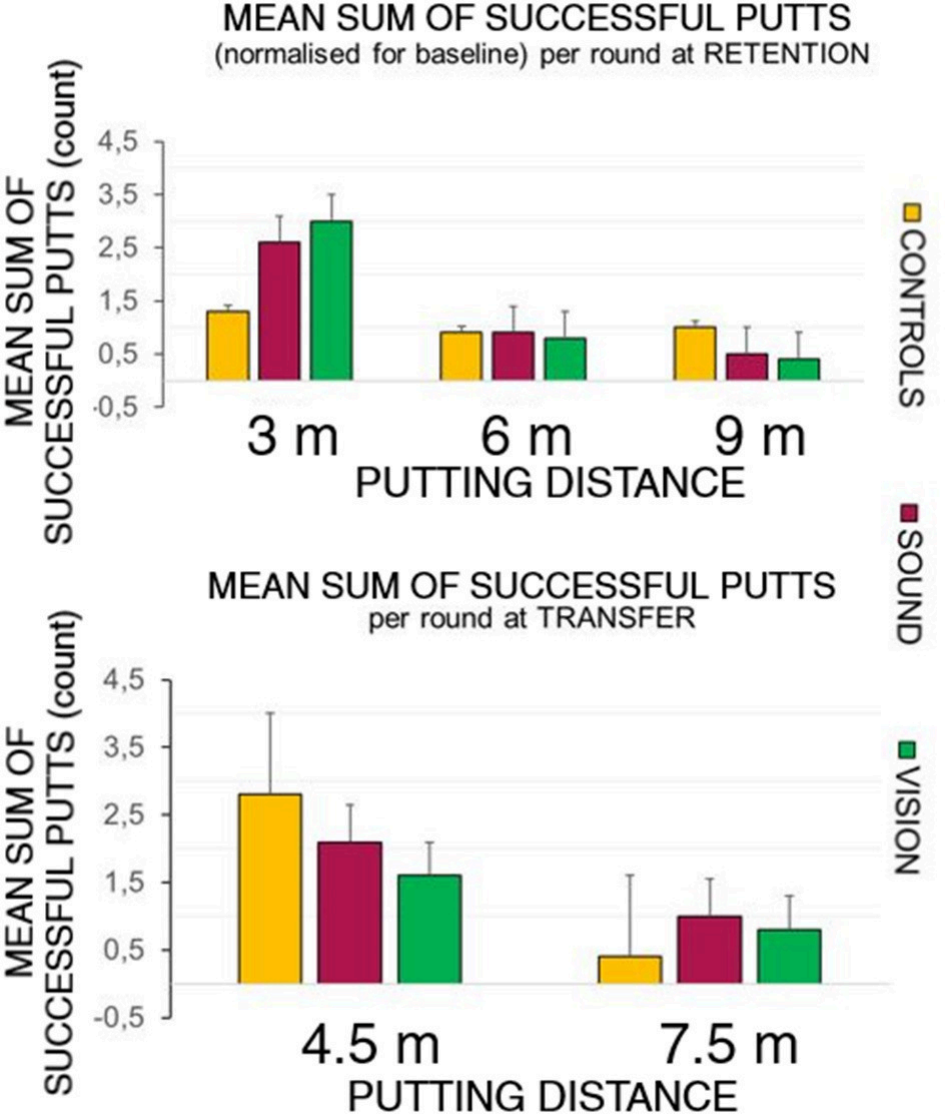
Success rates breakdown at retention and transfer across groups. The top panel depicts the sum of the average shot rate per round for the three groups: GC, GS, and GV. The sensory groups scored higher at the 3 m distance during the retention trials compared to the control group. The bottom panel shows the hit rates observed during the transfer test. The control group performed better than the sensory groups at the 4.5 m distance, but not at the longer 7.5 m distance.

Figure 5 top panel represents the overall number of successful putts (defined as ball going into the target hole) per group per round normalized for the baseline for the first five putts at the beginning of each learning session. The bottom panel represents number of successful hits for practice trials across learning sessions. Figure 6 illustrates performance of participants for transfer and retention sessions.

##### Retrieval trials (1:5)

In the In the retrieval trials, the first five trials (without any display for GS and GV), showed improvement across sessions when hitting to **3 m** compared to **6** and **9 m**. We found the following main effects: **learning session number** on the gain in success rate *F*_(7, 189)_ = 5.1, *p* < 0.01, η_*p*_^2^ = 0.16, main effect of **target distance** *F*_(2, 30.7)_ = 10.17, *p* < 0.01, η_*p*_^2^ = 0.28 and interactions: target distance^*^learning session number *F*_(14, 201.78)_ = 2.49, *p* = 0.01, η_*p*_^2^ = 0.08. Bonferroni corrected pairwise comparisons revealed significant differences (*p* < 0.05) between T1 (0.03 ± 0.01) and learning sessions T6 (0.11 ± 0.03), T7 (0.13 ± 0.02), and T8 (0.13 ± 0.16). There was a significant difference between performance at the target putt distance **3 m** (0.16 ± 0.03) and **9 m** (0.03 ± 0.01), *p* < 0.01, and between **6 m** (0.07 ± 0.01) and **9 m**, *p* < 0.01.

To further investigate this relationship, we looked at how the radial distance from the hole changed over sessions. This variable was derived using the coordinates of the final ball position and the origin of the hole in metric units and was normalized with respect to baseline data for each participant. **Figure 8** shows changes over the sessions for retrieval trials (top panel). In the retrieval trials we found significant main effects for putting **target distance** [*F*_(2, 54)_ = 6.12, *p* < 0.01, η_*p*_^2^ = 0.18], and **learning session number** on the distance from the hole [*F*_(4.2, 115.8)_ = 4.46, *p* < 0.01, η_*p*_^2^ = 0.14]. Bonferroni corrected pairwise comparisons revealed a significant difference within learning sessions T1 (0.93 ± 0.05) and T7 (0.74 ± 0.05), *p* = 0.02, and T1 and T8 (0.70 ± 0.06) *p* < 0.01. There was a significant difference between performance at target distance **3 m** (0.74 ± 0.07) and **9 m** (0.93 ± 0.05), *p* = 0.01, and between **6 m** (0.71 ± 0.05) and **9 m**, *p* < 0.01.

##### Practice trials (6:20)

In the practice trials that included fifteen putts to each target distance that directly followed retrieval trials, all groups improved with time, but the improvement in GS and GV was more pronounced. We found a main effect of **learning session number** at the hit rate *F*_(7, 189)_ = 9.09, *p* < 0.01, η_*p*_^2^ = 0.25, **target distance** *F*_(1.18, 30.7)_ = 38.18, *p* < 0.01, $\eta_{\text{p}}^{2}$ = 0.59 and interactions: **target distance** ^*^ **learning session number** *F*_(14, 201.78)_ = 2.49, *p* = 0.01, η_*p*_^2^ = 0.08, and target distance ^*^ learning session number^*^group *F*_(14.95, 201.78)_ = 2.17, *p* < 0.01, η_*p*_^2^ = 0.14. Figure 5 depicts a more pronounced increment in the success rates at **3 m** for GS and GV than for GC. Bonferroni corrected pairwise comparisons revealed significant differences (*p* < 0.05) between T1 (0.09 ± 0.01) and learning sessions T5 (0.15 ± 0.02), T6 (0.16 ± 0.02), T7 (0.18 ± 0.01), T8 (0.17 ± 0.02). There were significant differences (*p* < 0.01) between performance at target distance **3 m** (0.27 ± 0.03) and **9 m** (0.04 ± 0.01), and **6 m** (0.1 ± 0.01), and **9 m**. No group differences were found.

**Figure 7:**
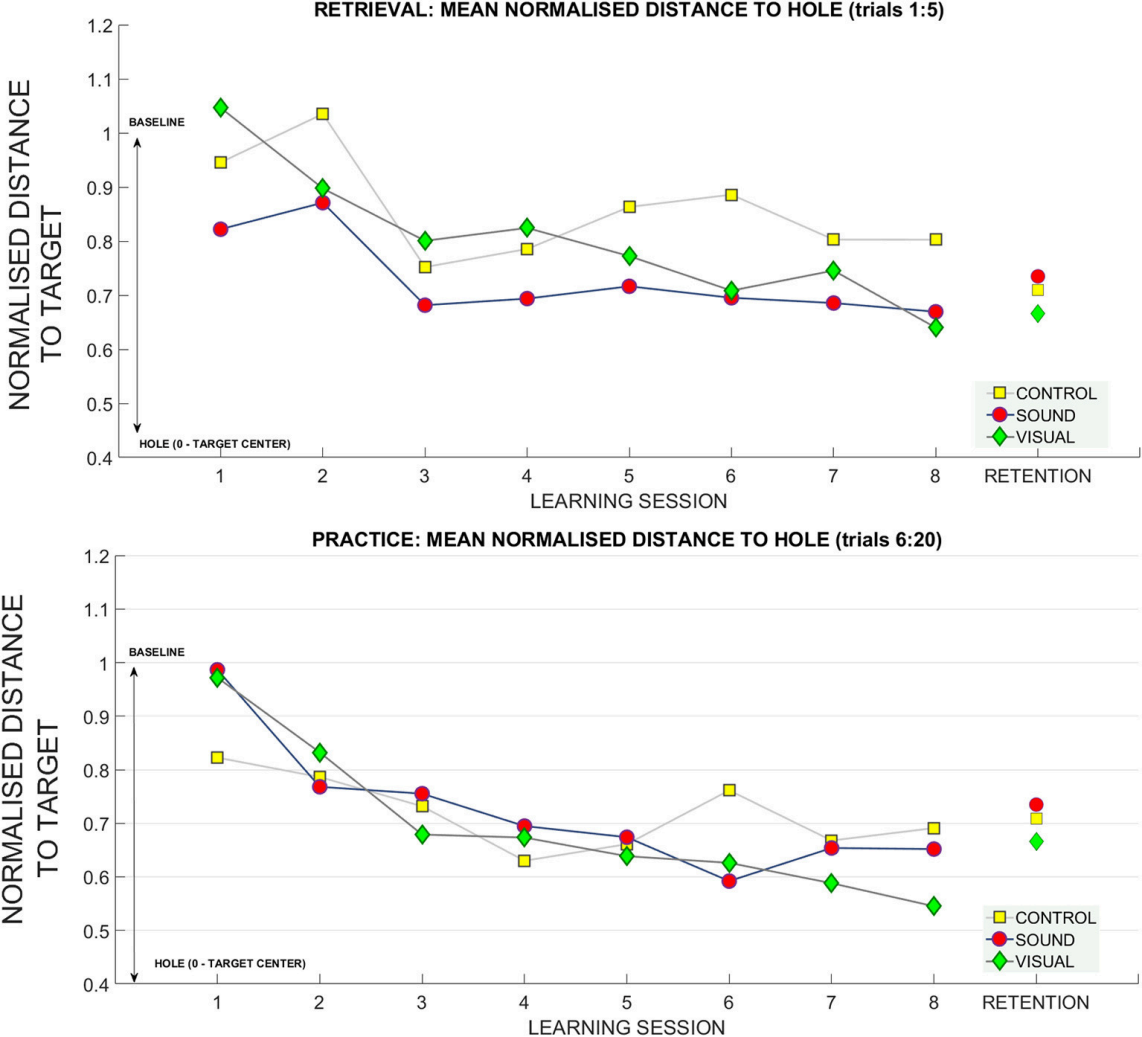
The gain in distance (calculated using Pythagoras's theorem) to the hole across the learning and retention conditions. The decrease in the average distance to the hole (normalized for baseline performance) for each subject (where 1 stands for performance equal to baseline, and 0 reaches the hole). The top panel depicts the retrieval trials (first five trials across each learning session) compared to retention. The bottom panel depicts practice trials (fifteen putts following retrieval trials) across each session compared to retention trials.

Figure 7 shows changes in radial distance from the hole over the sessions for practice trials (bottom panel). For practice trials we found significant main effects for **target distance** [*F*_(2, 54)_ = 13.6, *p* < 0.01, η_*p*_^2^ = 0.33] and **learning session** on ball distance to the hole [*F*_(4.06, 109.7)_ = 12.4, η_*p*_^2^ = 0.31] and also a significant interaction between **target distance** ^*^ **learning sessions number** ^*^ **group** [*F*_(12.74, 172)_ = 1.6, *p* = 0.03, η_*p*_^2^ = 0.11]. Bonferonni corrected pairwise comparisons revealed significant differences (*p* < 0.01) between T1 (0.93 ± 0.06) and learning sessions T3 (0.72 ± 0.04), T4 (0.67, ± 0.04), T5 (0.65 ± 0.05), T6 (0.65 ± 0.05), T7 (0.64 ± 0.03), T8 (0.63 ± 0.04). There was a significant difference between performance at target distance **3 m** (0.55 ± 0.07) and **9 m** (0.89 ± 0.04), p < 0.01, and **6 m** (0.69 ± 0.04) and **9 m**, *p* = 0.01. No group differences were found.

##### Retention

At the retention test (top panel of Figure 6) there was a significant effect of putt target distance on the number of successful putts normalized to baseline *F*_(1.39, 37.6)_ = 14.46, *p* < 0.01, η_*p*_^2^ = 0.35, with Bonferonni corrected pairwise comparisons demonstrating differences between on distances 3 m (0.24 ± 0.03) and 6 m (0.09 ± 0.02) rate *p* < 0.01, and 3 m and 9 m (0.06 ± 0.01) *p* < 0.01.

There was also a main effect change in radial distance to the target of **target distance** [*F*_(2, 4)_ = 12.90, *p* < 0.01, η_*p*_^2^ = 0.32], with differences between **3 m** (0.54 ± 0.04) and **9 m** *p* < 0.01 (0.91 ± 0.07) and between **6 m** (0.65 ± 0.05) and **9 m** (*p* = 0.01).

##### Transfer

The bottom panel of Figure 6 depicts performance at the transfer test in all groups putting to the 4.5 and 7.5 m distances. Due to a violated assumption of normality for the residuals we ran a Wilcoxon Signed Ranks Test, Z = −3.5, *p* < 0.01 for two conditions. A Kruskal-Wallis Test revealed no differences between groups in performance at the transfer test.

### Results Referring to Research Questions 2 and 3

#### Kinematic Variability (Impact Velocity)

We have pooled together all practice trials from all participants across all lengths and learning sessions (30 participants × 8 learning sessions × 3 distances × 15 practice trials for each distance) to verify if the key factor in kinematic performance that influenced the distance of ball traveled was impact velocity. We found, using a linear model, that impact velocity explained 82% (Adjusted R-Squared 0.82 *p* < 0.01) of the distance the ball traveled (measured as a function of putting metric distance). Therefore, to quantify the kinematic variability of performance across trials we extracted, for each participant, a standard deviation across trials [separately retrieval (1:5) and practice trials (6:20)] for impact velocity.

##### Retrieval trials (1:5)

**Figure 8:**
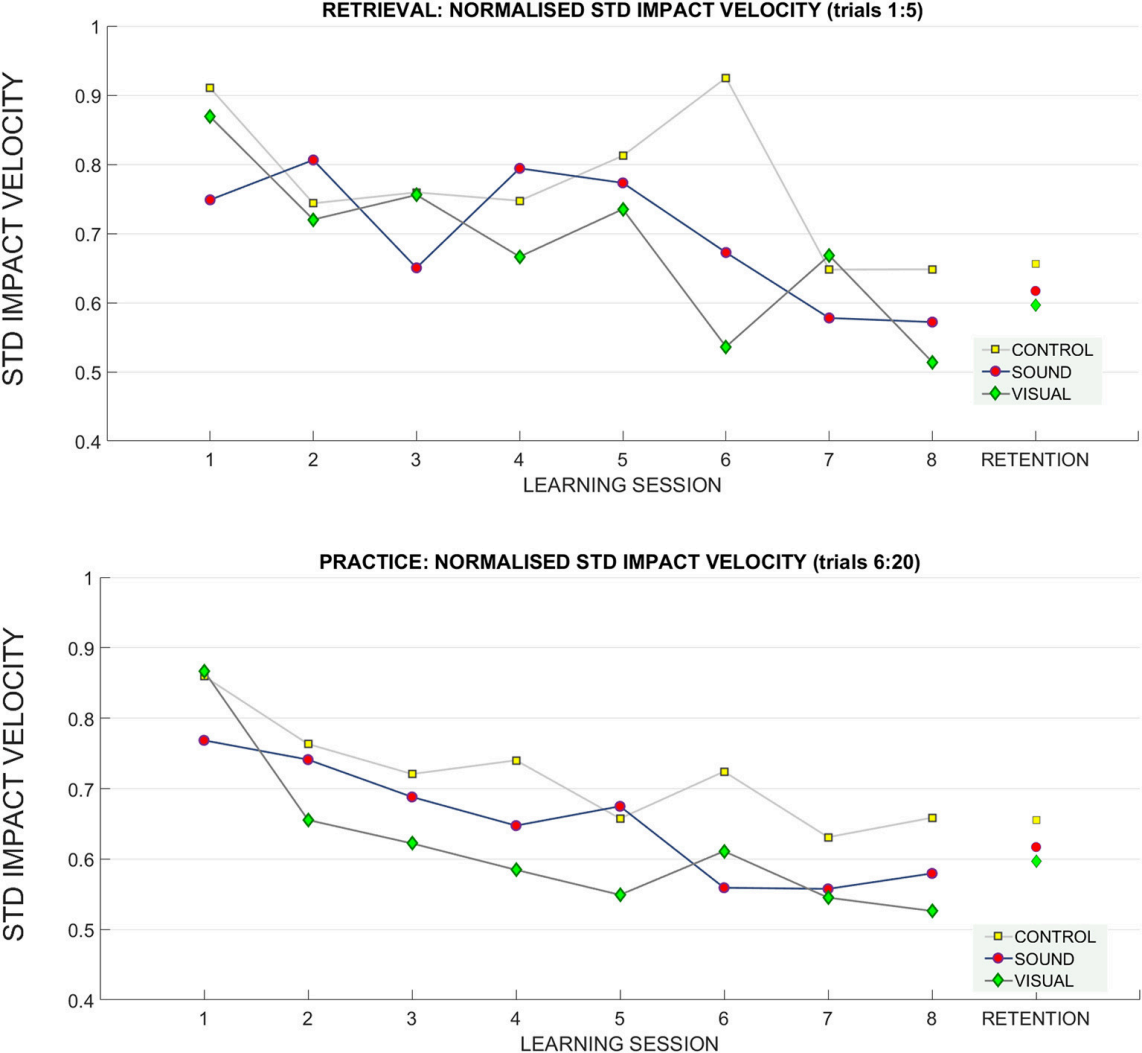
Changes in the standard deviation of the impact velocity across the learning and retention trials. A breakdown of the standard deviation of impact velocity across groups across sessions (**top**: retrieval trials, **bottom**: practice trials). During practice trials, the control group had a higher standard deviation of impact velocity than the sensory groups throughout training than both sensory groups, especially GV. There was no difference between groups at retention.

A significant main effect of **learning session number** on **impact velocity variability** (standard deviation) was found *F*_(4.80, 129.72)_ = 4.65, *p* < 0.01, $\eta_{\text{p}}^{2}$ = 0.15 in the retrieval trials (normalized to baseline performance), indicating some form of learning and skill acquisition associated with practice (see Figure 8 for reference). Bonferroni corrected pairwise comparisons revealed a significant difference within learning sessions T1 (0.84 ± 0.05) and T7 (0.62 ± 0.03), *p* < 0.01, and T1 and T8 (0.58 ± 0.03), *p* < 0.01.

##### Practice trials (6:20)

In the practice trials, the main effect **learning session number** on **impact velocity variability** was noted [F_(4.65, 125.76)_ = 10.06, *p* < 0.01, $\eta_{\text{p}}^{2}$ = 0.27). Main effect of interaction between T1 and T8 target distance^*^learning session^*^group on variability was found *F*_(12.74, 172)_ = 2.7, *p* = 0.04, η_*p*_^2^ = 0.16. Bonferroni corrected pairwise comparisons revealed significant differences (*p* < 0.05) between T1 (0.83 ± 0.04) and learning sessions T3 (0.67 ± 0.03), T5 (0.63 ± 0.04), T6 (0.63 ± 0.04), T7 (0.58 ± 0.02), T8 (0.59 ± 0.02). No other effects were found.

##### Retention

At retention there was a trend toward main interaction of **target distance** ^*^ **group** on **impact velocity variability** *F*_(2, 4)_ = 2.3, *p* = 0.07, η_*p*_^2^ = 0.14.

#### Timing Variability (Temporal Ratio)

In this section we present findings with reference to:

Professional players keep their temporal ratio between the duration of the backswing to forward swing constant across putts to different target distances. In our study we found that participants show a different pattern of behavior.

##### Retrieval trials (1:5)

**Figure 9:**
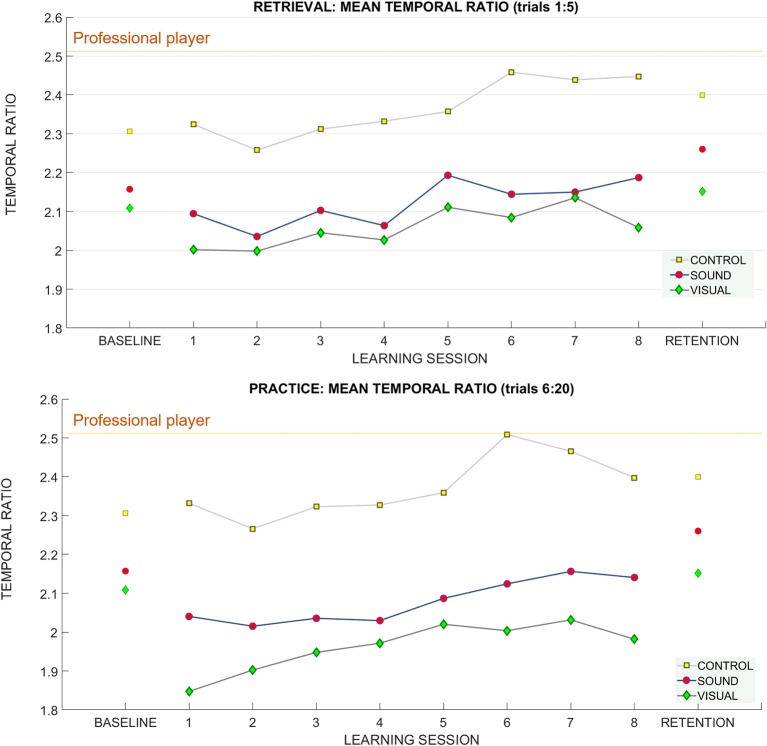
The temporal ratio (backswing duration/downswing duration) across learning and retention trials. Breakdown of the means for the temporal ratio across groups and across sessions (**top**: retrieval trials, **bottom**: practice trials). The mean of the temporal ratio for the professional player is demarcated with a purple horizontal line on both panels.

For retrieval trials—we found a significant effect of the putt **target distance on** the temporal ratio *F*_(1.4, 37.8)_ = 11.94 *p* < 0.01, η_*p*_^2^ = 0.3) suggesting that people adapted their putting timing pattern to accommodate different distances (see Figure 9). No learning session number or group effects were found. Bonferroni corrected pairwise comparisons revealed significant differences (*p* < 0.01) between the temporal ratios at all putt target distances **3 m** (2.1 ± 0.34), **6 m** (2.20 ± 0.39), **9 m** (2.26 ± 0.41).

**Figure 10:**
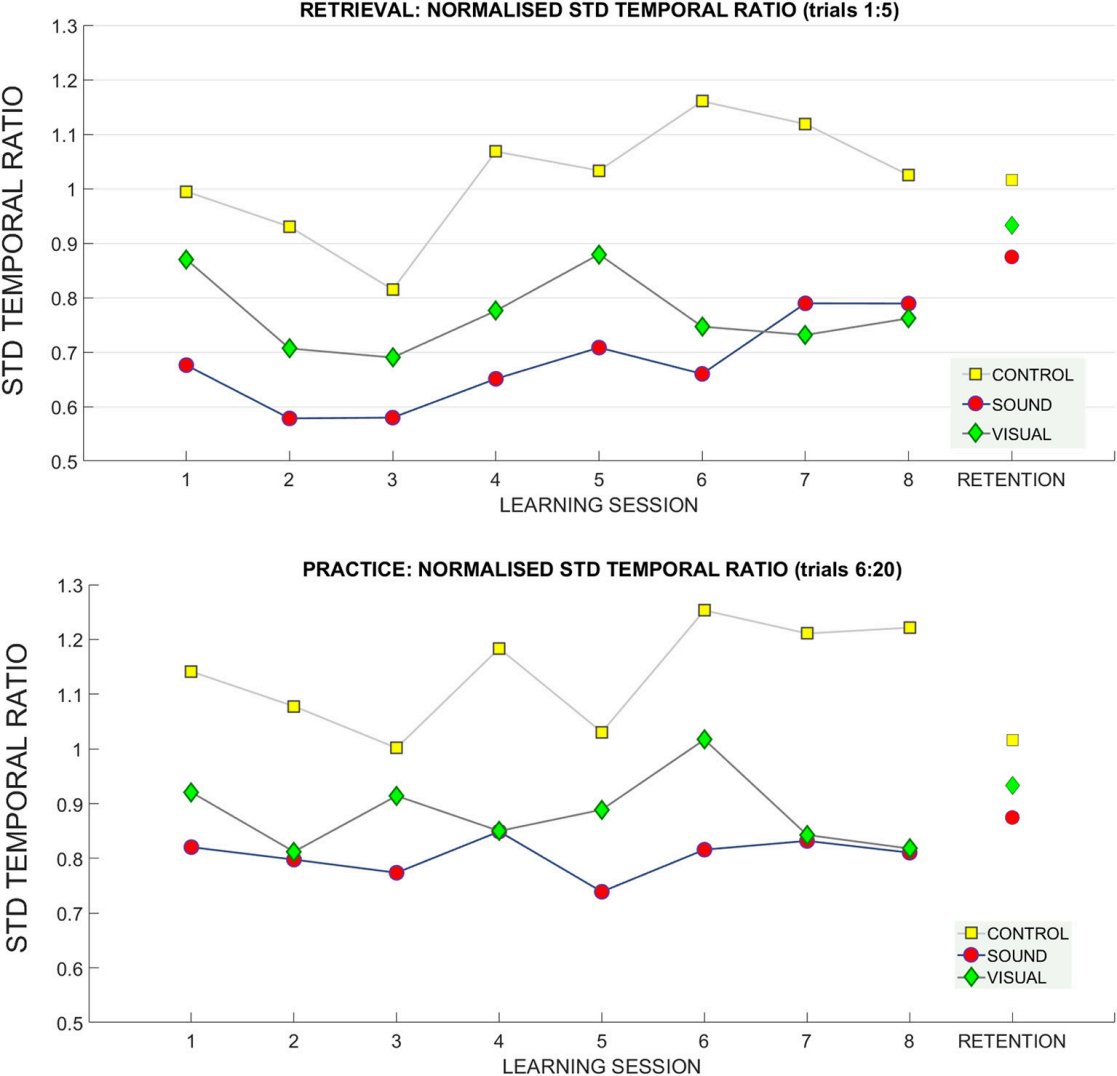
Standard deviation of temporal ratio across learning and retention. A breakdown of the standard deviation of the temporal ratio across groups and across sessions (**top**: retrieval trials, **bottom**: practice trials).

**For the standard deviation of the temporal ratio normalized to the baseline data** (Figure 10) we observed no main effects in the retrieval trials.

##### Practice trials (6:20)

For practice trials—we found a significant main effect for putt **target distance** on the temporal ratio [*F*_(2, 54)_ = 16.27 *p* < 0.01, η_*p*_^2^ = 0.37] again suggesting that people can adapt the putting timing pattern to achieve different putt distances (see Figure 9).

Bonferroni pairwise comparison found significant differences between temporal ratio for putt target distance between **3 m** (2.1 ± 0.34) and **9 m** (2.21 ± 0.43), *p* < 0.01, and **6 m** (2.15 ± 0.39) and **9 m**, *p* = 0.01.

We did find a significant interaction in the practice trials between **target distance** ^*^ **learning session number** ^*^ **group** [*F*_(10.09, 131.23)_ = 1.72, *p* = 0.01 η_*p*_^2^ = 0.11) and **standard deviation of temporal ratio** (see Figure 10). Bonferroni corrected pairwise comparisons did not reveal significant differences for any of the factors.

##### Retention

For retention we found a main effect of target distance *F*_(1.47, 39.8)_ = 8.22, *p* < 0.01, η_*p*_^2^ = 0.23 (see Figure 9). Bonferroni corrected pairwise comparisons revealed differences between **3 m** (2.20 ± 0.06) and **6 m** (2.27 ± 0.07), *p* = 0.01, and **3** and **9 m** (2.33 ± 0.08), *p* = 0.01.

No main effects or interactions were present for standard deviation of temporal ratio at the retention measurement (see Figure 10).

## Discussion

In this study, we wanted to investigate whether people can achieve better performance outcomes if a model template of the movement dynamics and tempo are made available to them through either an auditory or visual display. When compared to the performance of a control group, our data show that both groups exposed to sensory guidance showed improved task performance during learning.

Our first research question investigated whether novices can “learn” a golf putting task better when compared to a control group, where success is measured in terms of goal attainment. We found an interaction between groups at each learning session during the putts performed with assistance of sensory guides. However, those performance advantages were not present during the retrieval trials performed without sensory guides, or in the retention tests two weeks after the end of training sessions. We also did not find a difference between groups in the transfer test between trials.

With respect to our second research question, we wanted to see if the sensory guidance resulted in differences between the groups in terms of kinematic variability (standard deviation of impact velocity across trials) and timing variability (standard deviation of temporal ratio between backswing and downswing movement). We found significant interactions of group for both factors when putts were performed in the presence of sensory guides.

With regards to our third research question, we found that a sound guide that delivers the spatio-temporal characteristics of expert motion can influence the learning of a new, and complex motor task in a similar way to a visual display. This is particularly interesting considering that the acoustic display was representing information participants were not accustomed to having since they had no prior experience of golf putting. We did not observe differences between sensory guide groups in terms of performance suggesting that people were able to pick up information relating to the movement dynamics of a professional player from environmental sounds. This is consistent with Rosenblum et al.'s “Supramodal Brain Theory” (2017), mentioned in the introduction, according to which external events may be equally well perceived through visual or auditory channels, providing that the relevant information patterns are specified in either sense modality. However, the observed advantage compared to the control group was not significant in *post-hoc* tests.

Taken together, our findings suggest that sensory guidance during learning might lead to an enhancement of performance, but is limited to the presence of the guide. This phenomenon was previously described in the literature as sensory dependency (improvement present only when the guide is available) and has been reported in other studies (Anderson et al., 2005; Maslovat et al., 2009). We found the performance advantage was not retained 2 weeks after the end of training, with no specific transfer to other distances (**4.5** and **7.5 m**). Therefore, it seems that although a sound display improves real-time performance when learning a complex task, it does not carry over to performance in the absence of any sensory guidance. Below we will discuss important lessons that have been learned from this study and will suggest other ways in which sensory guidance could be used in a more practical and meaningful way to improve motor performance.

### Lessons Learned From the “Copycat” Approach

Auditory and visual guidance have been repeatedly reported in the literature to be efficient in modifying parameters of human movement in a directive way (Sigrist et al., 2013; Young et al., 2013; Schmitz and Bock, 2014; Danna et al., 2015; Effenberg et al., 2016; Bringoux et al., 2017). However, the majority of previous studies did not look into the use of sound guidance in a motor learning context. In our study, we confirmed that it can bring immediate benefits to performance, but we did not observe these benefits to be retained over time.

Our results suggest that the “copycat” approach we have explored in this study does not bring a long term advantage in performance when compared to learning without guidance. We see the issue regarding this observation as 3 fold. Firstly, sensory guidance has been demonstrated before to lead toward sensory dependency. Adams et al. (1972) described this as a “guidance hypothesis” and explained it as learners becoming over-reliant on the external sensory information and neglecting task-intrinsic, proprioceptive feedback. Therefore, when the guidance is no longer present (i.e., during retention tests) performance drops due to the underdevelopment of internal motor task representation during learning; caused by a neglect of proprioceptive feedback due to the attentional resources being deployed during sensory guidance (Anderson et al., 2005; Maslovat et al., 2009). In this respect, many researchers consider retention performance as a more accurate assessment of learning outcomes than the learning curve during training (Salmoni et al., 1984). The majority of the evidence in the literature about effectiveness of auditory signals in guiding motion comes from studies looking at concurrent real-time auditory feedback tracking parameters of a person's own movement, which is different from the “copycat” approach that tries to imitate the template of an expert's movement. For example, in a study looking at bimanual learning, Dyer et al. (2016) did not observe “guidance reliance” in an immediate retention test, with participants being better than controls when they previously trained with concurrent sonification feedback. The authors of this study hypothesized that extra auditory information might have enhanced the proprioceptive perception of the task goal timing pattern, rather than lead to the neglect of it. However, the observed advantage was diminished at the 24 h post-retention test. Dyer et al. (2017) postulate that the “guidance effect” can be avoided if sonification focuses on enhancement of the naturally occurring task feedback. This stance follows the proposal by Jacobs and Michaels (2007) that motor learning is in fact the training of attention to attend to streams of information that are relevant to task performance. In a similar vein, Buchanan and Wang (2012) demonstrated that if the feedback displayed is not juxtaposed spatially with the movement zone it does not hinder development of the spatial representation of the task. This does not only relate to visual guidance, but also auditory guidance. Arnott and Alain (2011) state that auditory pathway can feed information to action processing in the dorsal pathway (the headquarters of motor action guidance and navigating around space), especially with regards to directing attention to a designated space. Our results did not show any differences in retention between groups. Interestingly, the neuro-imagining study by Ronsse et al. (2011) in a concurrent feedback experiment suggested that the overreliance on visual guidance is stronger than auditory guidance, with the sensory areas being activated during task performance and decrease in auditory conditions. The design of that study, however, could not control for whether participants could memorize the task and the rhythm during practice, and this perhaps influenced their findings. We have found no evidence for this being the case in our study and we are also aware that the translation from studies using concurrent feedback to guidance paradigm (feedforward template of the expert's movement as in this study) is not straightforward. In our study, there was no difference at the retention phase between the performance of groups who used sensory guides when learning the task and those who did not.

Secondly, it is not completely clear how well humans can decode a kinematic template of movement from an auditory signal when it pertains to an environmental sound. Other studies have attempted to investigate the perception of biological motion in healthy adults using sound only (Murgia et al., 2012; Cesari et al., 2014; Kennel et al., 2014; Young et al., 2014). In our piloting phase (O'Brien et al., 2018) we demonstrated that people are able to distinguish between different speeds of golf swing via an auditory signal. This is in line with previous study of Murgia et al. (2012), which found that golfers can recognize their own swing motion via sound recording using two temporal parameters: temporal ratio and overall duration of the swing. Previous research in the visual domain has demonstrated that visual sensitivity to biological motion patterns seems to play a crucial function with links to cognition. For example, research has shown that there is a relationship between our ability to predict the outcomes of an unfolding of action and whether we have executed it before (Knoblich and Flach, 2001; Makris and Urgesi, 2015). Professional athletes demonstrated that they were able to distinguish whether a free throw shot was successful or not having only a point light display of the movement (Aglioti et al., 2008). In one study carried out by the authors, access to the visual point light display (depicting biological movement of healthy adults) resulted in the improvement of the temporal characteristics of an upper arm extension movement in a small sample of Parkinson's disease patients (Bienkiewicz et al., 2013). The brain activity unique for perception of such patterns has been identified by brain imaging studies to be a small area of the superior-temporal sulcus, more precisely the ventral bank of the occipital extent and a small region in the medial cerebellum (Grossman et al., 2000). This neurological circuitry is linked to the ability of animals to understand the action of others and imitate it (Rizzolatti and Craighero, 2004). Despite our reservations, both sensory groups developed their putting skills in a comparable fashion, suggesting that similar information was detectable through both the visual and auditory displays. This has implications for future studies investigating scenarios where sound might be a better fit for providing performance feedback as it is a portable, and relatively easy to implement as a stimuli, without burdening visual attention necessary to control spatial aspects of the task.

### Limitations

It should be noted that in our study, we did not test how the learned skills of golf putting would transfer to the performance on an actual putting green on a golf course. In addition, we are aware that participants in a non-lab setting would practice a more variable selection of shooting distances instead of **3**, **6**, and **9 m** during all trials. Also, running this experiment in a more ecological setting than a designated lab space could yield entirely different results. Therefore, the “copycat” approach in our laboratory study cannot be generalized to training in a real-life setting.

We also acknowledge that we did not test concurrent sonification in our study, but a feed forward movement template of sonified velocity of a professional player. This leads us to question whether velocity was the right parameter to sonify in this study. The current developments in our lab are focused on investigating motor learning with concurrent auditory feedback with different parameters of sound mapping. We hypothesize that different concurrent sonification methods could reinforce the proprioceptive feedback from movement and perhaps enhance learning to a greater extent than exposure to a template of the movement. In addition, both the sound and visual displays were artificially synthesized/engineered, which might have failed to convey the movement pattern as accurately as actual recordings of the movement (ecological sound, and/or video). Our analysis has been limited to a few of the variables that we deemed most interesting. In future research it is important to consider other factors that influence the precision of the golf ball's trajectory and speed: such as the face, loft and lie angles of the club, the location of impact on the club face (close to the “sweet spot”) along with the ratio of the shift of the center of pressure during the movement (Burchfield and Venkatesan, 2010).

## Author Contributions

MB wrote the manuscript, collected the data, and conducted the data analysis. FB was involved in setting up the experiment in the laboratory and reviewed the manuscript. MR, CC, LB and CB conducted conceptual work for this study and reviewed the manuscript.

### Conflict of Interest Statement

The authors declare that the research was conducted in the absence of any commercial or financial relationships that could be construed as a potential conflict of interest.

## Footnotes

^1^This is why the resulting velocity remains insensitive to the exact shape of the force profile, so long as the force remains rooted in the second harmonic of the resonance (Grober, 2009, p.22). The force applied in the backswing phase should equal, in magnitude, to the force applied in the downswing, with the length of the backswing defining the speed of impact.
